# Multisectoral and Inclusive Strategies for Improving Pregnant Adolescents’ and Teenage Mothers’ Access and Utilisation of Sexual and Reproductive Health Services in Kenya

**DOI:** 10.24248/eahrj.v8i3.797

**Published:** 2025-01-30

**Authors:** Claudia Robbiati, Rose Olayo, Rose Opiyo, Esther Waduu, Andrew Chemoiywo, Gloria Nacca, Alessia Ranghiasci, Silvia Declich, Maria Grazia Dente

**Affiliations:** a Italian National Institute of Health (Istituto Superiore di Sanità), Rome, Italy; b Masinde Muliro University of Science and Technology, Kakamega, Kenya; c The International Committee for the Development of Peoples (CISP), Nairobi, Kenya

## Abstract

**Background:**

Adolescent girls between 15 and 19 years of age make up just over one-fifth of the women of Kenya, and they account for 14% of all births. This study explored barriers to access and utilization of sexual and reproductive health services (SRH) for pregnant adolescents and teenage mothers in Kakamega County (Kenya).

**Methods:**

The qualitative study included a desk review, interviews and focus group discussions and a validation workshop with the engaged stakeholders to produce a framework for action.

**Results:**

The main barriers emerged in the following domains: acceptability (stigma and socio-cultural influences, negative healthcare workers attitude, lack of privacy and confidentiality), accessibility (distance to the health facility, costs for transport and drugs, opening times), availability (lack of staff, drugs and equipment, low coverage of SRH services specific for adolescents), contact/use (lack of information about SRH services offered), effectiveness (poor collaboration between all the relevant sectors and stakeholders, lack of adequate financing, no inclusion of adolescent perspectives in the policy-making process, lack of reliable data). Moreover, COVID-19 starkly impacted access and utilization of the services.

**Conclusion:**

A pathway to impact framework was coproduced building on the findings of the research to guide decision-makers in Kakamega and Kenya to improve access and utilization of SRH services for adolescents and especially pregnant girls and teenage mothers. Crucially, a multisector and multistakeholder approach including adolescent voices, could support the effectiveness of SRH services for adolescent girls.

## BACKGROUND

The World Health Organization defines an adolescent as any person aged 10–19 years and adolescents make up 1.2 billion (or 16%) of the world’s population.^1,2^ Adolescent sexual and reproductive health (ASRH) is a global health challenge, and mainly for adolescent girls that may experience unwanted sex or marriage, unintended pregnancies, and unsafe abortions.^3^ Studies have shown that women who become mothers in their teens are more likely to drop out of school and have reduced career progression and economic achievements, decision-making power and autonomy.^3^ Also, there is an increased risk of maternal and new-born deaths and disability.^4^

In Sub-Saharan Africa teenage pregnancy is a widespread emergency that needs to be addressed to reduce the health and socio-economic consequences that it entails.^5^ Almost 63% of pregnancies among adolescent girls in Kenya are unintended, with 35% ending up in abortion.^6^ Adolescent girls between 15 and 19 years of age make up just over one-fifth of the women of Kenya, and they account for 14% of all births.^7^ The coverage of health facilities offering adolescent friendly services is 62%, and more than half (53%) of the adolescent girls who give birth each year have less than four antenatal care visits and 31% do not give birth in a health facility.^8^ Teenage girls also are victim of sexual violence that is experienced by 15.6% of girls before the age 18 years in Kenya^9^ and adolescent girls account for 33% of the total number of new HIV infections in the country.^10^

The COVID-19 pandemic containment measures, especially the closure of learning institutions, has worsened the teenage pregnancy situation in Kenya, according to recent media reports and studies.^11,12^

Kenya’s government produced a broad policy framework about adolescent sexual and reproductive health. In 2003 the “Adolescent Reproductive Health and Development Policy” was Kenya’s first policy specifically addressing adolescents’ health.^13^ In 2005, the “National Guidelines for Provision of Adolescent Youth-Friendly Services” outlined essential ASRH services that adolescents should be able to access and how to improve their implementation, monitoring and evaluation.^14^ The “National Reproductive Health Policy” in 2007 promoted a multi-sectoral approach to address adolescents’ sexual and reproductive health needs and the right of adolescents to access information and youth-friendly health services.^15^ The “2010–2012 Reproductive Health Communication Strategy” underscored that young people should have access to appropriate information and health services.^16^ In 2015, the “National Adolescent Sexual and Reproductive Health Policy” promoted ASRH and an increased access to information and age-appropriate comprehensive sexuality education, with particular attention to vulnerable groups.^17^ Finally the 2019 “Kenya National Family Planning Guide” dealt with youth-friendly family planning services for adolescents.^18^ Despite the huge policy-making efforts of the Kenya government about ASRH, the implementation of such policies has been hampered by different types of barriers, resulting in an inadequate provision and access to ASRH services for adolescents, especially for the most vulnerable populations like pregnant girls.^19^

Kakamega county is located 360 km from Kenya’s capital city Nairobi. Kakamega is the second most populous county in Kenya with a population of 1,867,579 people with one in four people being an adolescent, and with a high burden of teen pregnancy of 19,4%.^20^ A previous study showed that for adolescents in Kakamega the access and utilization of health services remains a challenge and the main barriers include negative healthcare workers attitudes, distance to the health facility, unaffordable cost of services, adverse socio-cultural norms, lack of privacy and confidentiality in the health center.^19^

The purpose of this research study was to explore and synthesize available information about barriers to access and utilization of ASRH services for pregnant adolescents and teenage mothers, in Kakamega County and identify pathways for action.

## METHODS

### Desk Review

The desk review included a non-systematic search of the peer-reviewed literature and grey literature to gather information about ASRH services in Kenya and define the context of the research. The search was performed in PubMed, Google Scholar and Google on the 15th of June 2021 (no timeframe limitations were applied).

### Qualitative Investigation

The qualitative investigation was conducted in June-July 2022 in two Kakamega sub-counties of Matungu and Mumias West, chosen for their high teenage pregnancy rate, and included Focus Group Discussions (FGDs) and Key Informant Interviews (KIIs).

A total of 53 KIIs were conducted targeting key stakeholders with national and county institutions (3), sub-county institutions (11), international organizations and donors (2), helathcare and community health workers (15), civil society (1), community members such as community leaders, teachers, adolescent girls from support groups (21). The participants were invited to take part in the study through a formal contact by phone, email, letter or in person and the informed consent form was shared and discussed with them.

A total of 32 FGDs were conducted with adolescent mothers and pregnant adolescents (13), adolescent girls (6), family members (8) and Adolescent fathers (5). The recruitment was done through community engagement activities and the informed consent was read and explained to the participants. The FGDs were held separately for each category of beneficiary and lasted around 60 to 90 minutes.

A two-day training workshop was arranged to provide guidance to the research team composed of researchers from Masinde Muliro University of Science and Technology (Kenya), about the study tools and procedures and how to apply them in the field. The emphasis of the training was on qualitative data collection techniques and reporting, and ethics principles with a special focus on pregnant adolescents and teenage mothers access to ASRH services. A pre- and post-test was conducted to assess the level of knowledge and understanding. Pilot testing was performed with communities not participating in the study, to improve the acceptability and feasibility of the research approach and tools. For the piloting two FGDs were undertaken with groups of adolescent mothers and pregnant adolescents. Additionally, KIIs were conducted with the nurse in charge of reproductive health, two village elders and two community health workers (CHWs). The results of the piloting were fed into the revision of the tools (language, wording, sequentially, and relevance). The study received ethical approval from Masinde Muliro University of Science and Technology (PKU/2342/E1481) and the research licence from the National Commission for Science, Technology and Innovation (NACOSTI/P/22/16611).

### Validation Workshop

On the 17th of November 2022 a 3 hour workshop has been organised in Kakamega town and 53 attendees joined from Ministries, Government, health system, research institutions, civil society and media. The workshop adopted a participatory approach to discuss the research results and co-produce pathways for action to improve access and utilization of ASRH services for pregnant adolescents and teenage mothers.

### Conceptual Framework

**Figure 1.**
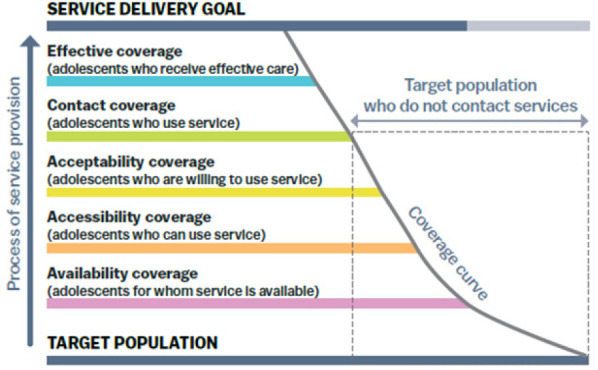
The Five Dimensions of the Tanahashi Framework

The study was primarily grounded in the Tanahashi framework health service coverage.^21^ The Tanahashi framework includes the following dimensions (Figure 1): availability, accessibility, acceptability, contact/use and effective coverage.

From “Handbook for conducting an adolescent health services barriers assessment (AHSBA) with a focus on disadvantaged adolescents. Geneva: World Health Organization; 2019”.^21^

## RESULTS

Documented The desk review retrieved 13 documents (7 reports and 5 peer-reviewed articles) highlighting barriers to ASRH friendly services encompassing the acceptability, availability, accessibility, contact/use dimensions of coverage of the services. No information was retrieved for the effectiveness dimension. Only one research study performed in Nairobi highlighted some facilitating factors related to the acceptability and accessibility dimensions. The main barriers emerging from the literature review are reported in Table 1.

**Table 1.** Main Barriers Emerging from Desk Review

Dimension	Main barriers
Availability	Low coverage of ASRH services, few HCWs are equipped with the necessary knowledge & skills, lack of commodities & supplies & dedicated spaces.
Accessibility	Long distance, high costs and poor availability of transport, long waiting times and inconvenient hours of the services, high cost of the services.
Acceptability	Socio-cultural norms and stigma, negative attitudes of HCWs, poor gender-responsiveness, poor age-appropriateness, perceived poor quality of services, limited self-efficacy and decision-making power of adolescent girls, & perceived discrimination by adults including HCWs.
Contact/use	Lack or limited awareness about ASRH services provided and their location, incorrect knowledge of adolescents about their sexual and reproductive health needs.
Effectiveness	No information was retrieved.

### The qualitative investigation

The qualitative investigation explored the five dimensions of the Tanahashi framework related to access and utilization of health services for pregnant adolescents and teenage mothers, the COVID-19 impact and priority actions to enhance ASRH services.

### Availability coverage

Lack of drugs, equipment, and doctors are the main barriers described for the availability dimension. Few HCWs are equipped with the knowledge and skills to provide adolescent friendly services and they often face heavy workloads and competing priorities. Lack of dedicated spaces for adolescents is also limiting their access and utilization of ASRH services. Perception of coverage of youth friendly ASRH services is generally low among adolescent girls.

*“I feel that the majority of adolescents do not attend services because we don’t have room for youth, like the youth friendly services they are supposed to be activated”* (HCW)

*“They are trying but they are not open and free. The private questions they should ask they will just ask you in an open environment so everyone hears….”*(Adolescent Mother)

### Accessibility coverage

Long distances to health facilities offering ASRH services, high costs and availability of transport are limiting the possibility for teenagers to access these services. Long waiting times, inconvenient hours of the services, lack of ambulance service are barriers to their accessibility. Adolescents can’t access health services also because they are asked to pay for services or medications, or to have parental consent to receive contraceptive methods (if they are under 18 years of age).

“Y*ou pay 500 shillings so that the patient is registered before being treated. If you don’t pay for registration, there will be no service”*(Adolescent father)

*“So, I was told to go buy two sets of gloves, yet I had nothing. I explained to the nurse and she told me to go away”*(Teenage mother)

*“If they are under 18 years of age they need parent consent to receive contraceptive methods”* (CHW)

The negative attitude and behaviour of some health services providers, like unfriendly and judging behaviour, was frequently reported as a main barrier to health services accessibility.

*“The first challenge is harsh speech. I think we have a challenge with the nurse’s language.”* (Adolescent Mother)

Some adolescents identified the inconvenient opening hours of the health facility clinics as a hindrance to adolescents accessing health services. Adolescents complained that the opening hours of the health facilities are not followed as indicated.

*“The problem is that some nurses come in late and they don’t observe arrival protocol and when its time they send you back home. If it’s you, will you go back to the clinic? I cannot go back”*(Adolescent Mother)

Adolescents mentioned that their boyfriends were afraid of the advice the girls would have received from the health care providers.

*“Dating a boyfriend who has negative attitude towards adolescent sexual health reproductive services is a factor. They perceive that once you go for the services you might come back having changed your mind about the relationship”*(Adolescent mother)

### Acceptability coverage

The most common barriers for pregnant adolescents and teenage mothers to access reproductive health services were socio-cultural norms widespread in the communities.

Generally, knowledge and utilization of these services are viewed as a sign of sexual promiscuity and are resisted by families and community members. Adolescent girls face stigma and discrimination, they fear to be seen by people they know while accessing reproductive health services and they are afraid of judgment and negative consequences.

*“Most of the doctors here are from this village and even if you are alone with him and he tests you and finds that you have got a disease, the issue will just get to the village and you will feel demoralized”* (Adolescent Mother)

*“They are usually looked down upon by everyone and they are seen as if they have already messed up their lives”* (Teacher)

*“The belly is protruding so you fear walking. So sometimes I just go say am going to the clinic and I sit in a certain bush then I come back home”* (Pregnant girl)

Sexual and reproductive health is not a topic discussed between parents and adolescents because it is a cultural taboo, and discouraged the use of ASRH services.

*“Sometimes we fear our parents. Some parents are strict and unable to talk to us because the culture does not allow hence we fear sharing such information with them. My mum realized I was pregnant just before I delivered.”* (Adolescent girl)

Some cultural misconceptions act against sexual and reproductive health services utilization.

*“Some believe when you use family planning you will not bear child later on in life, some believe that when you use a condom you will have some stomach-ache”*(Adolescent girl)

### Contact/Use Coverage

Lack or limited awareness about ASRH services provided and their location, and the incorrect knowledge of adolescents about their sexual and reproductive health needs, are limiting their contact and use of these services. Health-seeking behaviour among adolescents is also compromised by fear of questions and cultural or religious beliefs.

*“There are churches that forbid going to the hospital. They believe the Lord is the creator and protector of the baby”* (Family member)

### Effectiveness Coverage

Poor coordination and collaboration among sectors and inadequate financing, no inclusion of the adolescent perspective in the policy-making process and no reliable data about services utilization were described as barriers related to the effectiveness dimension.

*“Programs and stakeholders do not collaborate even if they are addressing the same issue”* (Government stakeholder)

*“Girls would go to this clinic today, and because of the stigmatization, they will move to another and another clinic. So that sequence could not be followed because they may not use the card they used in the previous clinic”* (Health officer)

### COVID-19 Impact

COVID-19 pandemic starkly impacted the access to health services for the adolescent girls. One of the main barrier described was that masks were requested to enter the health facilities; however the girls couldn’t afford to buy them or masks weren’t available on the market. Also curfew, travelling restrictions, long waiting time and lack of money hampered accessibility to the health services. Fear of contagion and social distance between the girls and the HCWs meant less privacy. COVID-19 also caused loss of employment, less money available, less food available, increased cost of life, school closure and deaths in the community.

*“Before you could afford soap at twenty shillings currently you cannot”*(Community organisation)

*“During the pandemic budget dedicated to health programs have been reallocated for the COVID-19 response”* (Government stakeholder)

School closures brought to an increase in sexual activity among adolescents, and inaccessibility of ASHR services and contraceptives brought to an increase in the number of pregnancies and school dropouts.

*“So when we were recalled to schools, many didn’t come back because some were nurturing children, others were almost giving birth, others were in hospitals nearing birth. And other even wrote their national exams while in hospital”* (Teacher)

*“The COVID-19 pandemic showed us that schools have a preventive role against adolescent pregnancy”* (Government stakeholder)

### Priority Actions to Enhance the Coverage of ASRH Services

*“The first thing is that they need to understand us and know our needs”* (Adolescent girl)

A common improvement suggested is to increase the number of HCWs, the availability of drugs, equipment and ambulances, and to extend opening time. Also increase HCWs salary could avoid strike and disruption and increase motivation. At the same time, HCWs need to be trained about ASRH and how to deliver adolescent friendly services. Adolescent dedicated spaces and a special day of attendance could improve privacy and make girls feel at ease. Support girls with transport costs could also facilitate their attendance.

*“There should be a special day for the pregnant adolescents and teenage mothers where they attend their clinical services to enable all of them to attend to the services without fear”* (Community Chief)

Reaching adolescents within communities is also pivotal, mobile clinics could visit each community in coordination with CHWs, health facilities and schools and increase awareness of adolescents, family and community members.

“*Mobile clinics should be considered so that they can visit each area”* (CHW)

Schools have also a pivotal role in educating adolescents and their family and to contribute together with community leaders to fight stigma about ASRH. Engaging adolescents who went through the same experience could enhance this process. Schools need to be inclusive and support pregnant adolescents and teenage mothers in their educational journey.

*“Some schools restrict them, like they did not give them a chance to study when they’re pregnant”* (Teacher)

A major progress would be to enhance coordination and collaboration at community and governance level, among different sectors and stakeholders. At community level good networking between the stakeholders like the CHWs, the HCWs, private clinics, and teachers could support adolescent girls to access ASRH services. At governance level an enabling environment supporting a multi-program approach, where relevant sectors like reproductive health, education, children protection work together by sharing data and pursuing common objectives, was deemed crucial.

*“An enabling policy framework supported by collaboration among different sectors and stakeholders is crucial for implementing effective policies”* (International Organization Representative)

*“Horizontal programs, for example adolescent health and school health, should work together, harmonizing resources and objectives”* (Government stakeholder)

Also, the quality of the collected data needs to be enhanced, in order to rely on updated and sound information that could help in the planning, monitoring, and evaluation of the activities.

### Stakeholder Workshop

The salient points that emerged from the discussion with the study stakeholders during the validation workshop are included in Table 2 and were then elaborated to develop the action framework to support access and utilization of ASRH services in Kenya.

**Table 2.** Main Cross-Cutting Actions Emerged During the Validation Workshop

Dimensions	Cross cutting actions
Availability	- Promote intersectoral collaboration between all the different actors involved in ASHR;
Accessibility	- Adapt the policy framework to be more attentive to the girls needs and include community perspectives;
Acceptability	- Consider adolescent health in preparedness measures to be as more inclusive and equitable as possible;
Contact/use	- Support adequate financing to continuously supply commodities to health facilities;
Effectiveness	- Train HCWs and empower CHWs to act as an effective interface between communities and health facilities;
	- Engage communities about ASHR, leveraging on CHWs and using participatory approaches;
	- Enhance awareness of girls about their health needs and health services available (school health programs, peer to peer education, girls’ groups).

## DISCUSSION

The purpose of this research study was to explore and synthesize available information about barriers to access and utilization of ASRH services for pregnant adolescents and teenage mothers, in Kakamega County and produce a framework for action. The results of this research study suggest that the main barriers to access and utilisation of youth friendly ASRH services are related to the following Tanahashi dimensions: acceptability (stigma and socio-cultural influences, negative HCWs attitudes, lack of privacy and confidentiality), accessibility (distance to the health facility, costs for transport and drugs, opening times), availability (lack of staff, drugs and equipment, low coverage of SRH services specific for adolescents), contact/use (lack of information about SRH services offered), effectiveness (poor collaboration between the relevant sectors and stakeholders, lack of adequate financing, no inclusion of adolescent perspectives in the policy-making process, lack of reliable data). It is therefore compelling to develop and implement interventions to improve ASRH services that address these components.

As the Lancet Commission on Adolescent Health and Well-being has suggested, “the most powerful actions for adolescent health and wellbeing are intersectoral, multilevel, and multi-component”.^22^ Interestingly the crucial role of collaboration among sectors and stakeholders to promote multisectoral interventions was highlighted during the interviews and the validation workshop. Thus, the action needs to involve different governmental and societal actors at different levels and from the relevant sectors. A multisectoral approach might entail some challenges such as effective coordination and communication, data sharing, common procedures, budget allocation etc., that would need to be considered. The multisectoral and multistakeholder approach would need to involve also the adolescent perspectives in the policy-making process and in the design and implementation of interventions.

The training of HCWs about welcoming and non-judgmental attitudes, privacy and confidentiality and the importance of reliable data collection, along with services enhancement in terms of equipment and drugs availability would be pivotal. Also, a community behaviour change intervention involving schools, communities, religious leaders and mass media should be promoted by peer educators and teachers.

This study also highlighted that during emergencies, like the COVID-19 pandemic, adolescent health would need to be carefully addressed to prevent undesired pregnancies, sexually transmitted infections, violence and school failure.^12^ Therefore, when designing pandemic and emergency preparedness strategies a special attention needs to be focused on vulnerable groups, such as adolescent girls and pregnant adolescents, to support access to health services (e.g. distributing masks to patients accessing health services) and schools.

**Figure 2.**
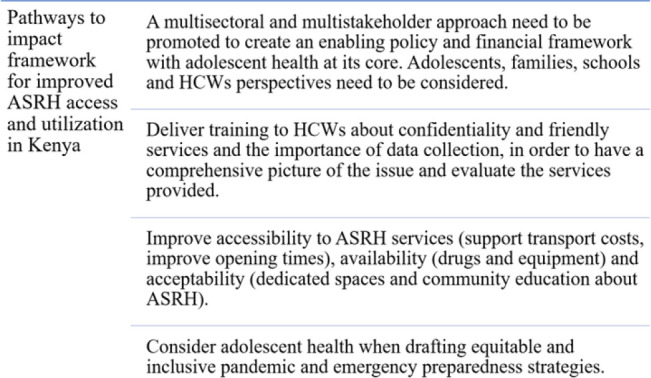
Pathways to Impact Framework

The pathways to impact framework reported in Figure 2 contains priority actions that the Kenya decision-makers should take into consideration to improve access and utilization of ASHR services.

## CONCLUSION

Adolescent pregnancy in Kenya and in Kakamega county is a public health and socio-cultural emergency. The results of this research suggest that a multisector and multistakeholder approach combining different programs, expertise and voices, could support adolescent pregnancy prevention and access to health services. A more inclusive approach that engages communities and increases awareness about ASRH and the training of HCWs about friendly services would also be pivotal. Improved accessibility, availability and acceptability and evaluation of these services are needed. Adolescent health has to be considered in strategies for pandemic and emergency preparedness to avoid unwanted consequences on health and education.

The pathways to impact framework produced should guide decision-makers in Kakamega and in Kenya to support access and utilization of health services for adolescents and especially vulnerable groups like pregnant girls and teenage mothers. Future research would need to validate the results in other areas of Kenya and evaluate the feasibility of the action framework.

### Limitations

The study included only two sub-counties and adopted a purposive sampling strategy. However, the two studied sub-counties have a high number of teenage pregnancies and the research team managed to engage in the study representatives of all the relevant stakeholders.
